# Dimethyl fumarate modulates M1/M2 macrophage polarization to ameliorate periodontal destruction by increasing TUFM-mediated mitophagy

**DOI:** 10.1038/s41368-025-00360-0

**Published:** 2025-04-17

**Authors:** Liang Chen, Pengxiao Hu, Xinhua Hong, Bin Li, Yifan Ping, ShuoMin Chen, Tianle Jiang, Haofu Jiang, Yixin Mao, Yang Chen, Zhongchen Song, Zhou Ye, Xiaoyu Sun, Shufan Zhao, Shengbin Huang

**Affiliations:** 1 Institute of Stomatology, School and Hospital of Stomatology, Wenzhou Medical University, Wenzhou, China; 2 Department of Prosthodontics, School and Hospital of Stomatology, Wenzhou Medical University, Wenzhou, China; 3 Department of Periodontology, Shanghai Ninth People’s Hospital, Shanghai Jiao Tong University School of Medicine, Shanghai, China; 4 College of Stomatology, Shanghai Jiao Tong University, Shanghai, China; 5 National Center for Stomatology, National Clinical Research Center for Oral Diseases, Shanghai Key Laboratory of Stomatology, Shanghai, China; 6 Applied Oral Sciences and Community Dental Care, Faculty of Dentistry, University of Hong Kong, Hong Kong, China; 7 Department of Periodontology, School and Hospital of Stomatology, Wenzhou Medical University, Wenzhou, China; 8 Department of Oral Maxillofacial Surgery, School and Hospital of Stomatology, Wenzhou Medical University, Wenzhou, China

**Keywords:** Proteasome, Mitophagy, Periodontitis, Mechanisms of disease, Preventive medicine

## Abstract

Periodontitis is a common oral disease characterized by progressive alveolar bone resorption and inflammation of the periodontal tissues. Dimethyl fumarate (DMF) has been used in the treatment of various immune-inflammatory diseases due to its excellent anti-inflammatory and antioxidant functions. Here, we investigated for the first time the therapeutic effect of DMF on periodontitis. In vivo studies showed that DMF significantly inhibited periodontal destruction, enhanced mitophagy, and decreased the M1/M2 macrophage ratio. In vitro studies showed that DMF inhibited macrophage polarization toward M1 macrophages and promoted polarization toward M2 macrophages, with improved mitochondrial function, inhibited oxidative stress, and increased mitophagy in RAW 264.7 cells. Furthermore, DMF increased intracellular mitochondrial Tu translation elongation factor (TUFM) levels to maintain mitochondrial homeostasis, promoted mitophagy, and modulated macrophage polarization, whereas TUFM knockdown decreased the protective effect of DMF. Finally, mechanistic studies showed that DMF increased intracellular TUFM levels by protecting TUFM from degradation via the ubiquitin-proteasomal degradation pathway. Our results demonstrate for the first time that DMF protects mitochondrial function and inhibits oxidative stress through TUFM-mediated mitophagy in macrophages, resulting in a shift in the balance of macrophage polarization, thereby attenuating periodontitis. Importantly, this study provides new insights into the prevention of periodontitis.

## Introduction

Periodontitis, a chronic inflammation that invades periodontal tissue, is characterized by periodontal pocket formation, pocket wall inflammation, absorption of the alveolar bone, and gradual tooth loss.^1,2^ Clinical studies have shown that periodontitis is the sixth most prevalent disease worldwide and is currently the main cause of tooth loss in adults.^3^ Current treatments for periodontitis focus on dental calculus plaque removal and antimicrobial therapy without achieving satisfactory therapeutic results.^4^ With an increased understanding of periodontitis pathogenesis, modulating the immune response is an effective strategy for its prevention or treatment.^5^

Evidence has shown that macrophage polarization plays a crucial role in the innate immune response and progression of periodontitis.^6^ Studies have shown that macrophages stimulated by pathogen-associated and damage-associated molecular patterns polarize to M1 macrophages and undergo glycolysis, leading to mitochondrial dysfunction and the consequent aggravation of periodontitis.^6,7^ Moreover, the mitochondrial dysfunction caused by various stimuli impaired the transition of M1 to M2 macrophages, which are anti-inflammatory cells that facilitate periodontal tissue repair.^8,9^ However, when mitochondrial function is restored, transforming M1 into M2 macrophages is repaired.^9^ Our previous studies revealed the important role of mitochondrial dysfunction in developing periodontitis and found that improving mitochondrial function effectively inhibits mitochondrial dysfunction and promotes mitochondrial biogenesis, alleviating periodontitis.^10–14^ Mitochondria produce clear reactive oxygen species (ROS). During mitochondrial dysfunction, the balance between ROS in cells and mitochondria is disrupted, causing oxidative stress.^15^ Oxidative stress contributes to M1 polarization, and using antioxidant agents can effectively alleviate the classic activation of macrophages.^16,17^ Therefore, restoring mitochondrial dysfunction and inhibiting oxidative stress to modulate macrophage polarization may be a potential approach to prevent periodontitis.

Mitochondria are easily damaged, leading to mitochondrial dysfunction and oxidative stress; therefore, damaged mitochondria need to be separated from healthy mitochondria and removed, a phenomenon called mitophagy.^18,19^ Maintaining mitophagy plays an important role in mitochondrial homeostasis and ROS balance. Additionally, the physiological mechanism of macrophage activation includes inhibiting mitophagy, whereas inducing mitophagy can promote M1 macrophage polarization to M2 macrophages.^20,21^ According to recent studies, the activation of mitophagy leading to the restoration of mitochondrial function is essential in periodontitis prevention and treatment.^22,23^ A recent study showed a significant reduction in mitophagy-related protein expression, Pink1 and Parkin, in mouse models of periodontitis, suggesting that impaired mitophagy affects the development of periodontitis.^24^ Zhai et al. found that novel “mitochondrial nanorepairers” and mitochondria-targeting nanoparticles showed therapeutic effects against periodontitis by promoting mitophagy and removing damaged mitochondria in mesenchymal stem cells.^25^ Therefore, we suggest that targeting mitophagy is an effective method for treating periodontitis by inhibiting mitochondrial dysfunction and oxidative stress. TUFM is a class of proteins synthesized by mitochondrial DNA transcription.^26^ Recent studies revealed that TUFM regulates mitophagy and exerts anti-inflammatory and antioxidant effects.^27–29^ Given the strong link between TUFM and mitochondrial homeostasis, we suggest that TUFM plays a key regulatory role in periodontitis development.

DMF is a methyl ester of fumaric acid registered for treating relapsing forms of multiple sclerosis and psoriasis.^30^ DMF has been tested in several clinical trials for its anti-inflammatory properties and is currently used for treating patients with relapsing-remitting multiple sclerosis.^31^ DMF has excellent antioxidant and inflammatory properties, making it a potential medicine for treating periodontitis.^32,33^ Moreover, DMF confers neuroprotection by enhancing mitophagy in patients with Parkinson’s disease.^34^ It is worth noting that DMF has shown the ability to inhibit osteoclast differentiation by the Nrf2 activation and the decreased release of high mobility group box 1 (HMGB1) in vitro.^35–37^ In addition, recent studies have shown that DMF application inhibits classical macrophage activation, indicating that DMF may be a potential therapeutic agent for preventing periodontitis.^38–40^

We aimed to investigate the preventive effects of DMF on periodontitis and explore its underlying mechanisms.

## Results

### DMF ameliorates periodontal tissue destruction and modulates M1/M2 macrophage polarization in vivo

**Fig. 1:**
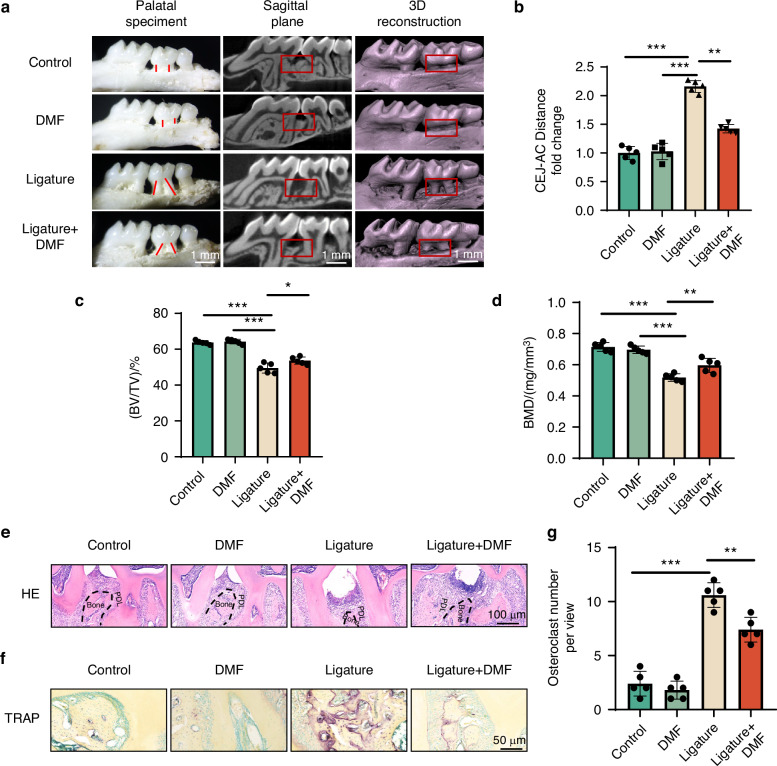
DMF ameliorates periodontal tissue destruction. **a** Macroscopic aspects of the maxilla in mice from control, DMF, ligature, and ligature+DMF groups with volume microscope and 3D reconstruction with Micro-CT. The red lines showed the distance from ACJ to the AC. Representative Micro-CT digitalis images of maxilla alveolar bone surrounding the second upper molar. The square frame showed visual differences in alveolar bone levels between the three groups. **b** Quantitative analysis of ACJ-AC distance (normalized to the Control group). **c** Quantitative analysis of bone volume over total volume. **d** Quantitative analysis of bone mineral density. **e**, **f** Representative images of hematoxylin and eosin and tartrate-resistant acid phosphatase stainings of the periodontitis areas. **g** Quantitative analysis of osteoclast number per view at alveolar bone. Data are presented as mean ± standard error of the mean. *P < 0.05, **P < 0.01, and ***P < 0.001 by one-way analysis of variance followed by Tukey's post hoc test. PDL periodontal ligament

Mice with periodontitis and healthy mice were administered^41^ DMF or saline daily to determine whether DMF protects against ligature-induced periodontitis.^41^ Compared with the control group, the ligature group had a significantly larger the cemento-enamel junction to the alveolar crest (CEJ-AC) distance, indicating that our experimental periodontitis mouse model was successful. After administering different DMF concentrations to mice, the CEJ-AC distance was reduced compared with that in the ligature group, and alveolar bone loss in mice administered 150 mg/kg DMF showed the most significant improvement. Therefore, the P + DMF 150 group was selected for subsequent analyses (Fig. 1a, b and S1a, b). Moreover, micro-CT results showed that the bone volume fractions (BV/TV) and bone mineral density (BMD) of the Ligature+DMF group were significantly higher than those of the ligature group (Fig. 1a, c, and d). These results suggest that DMF effectively reduces periodontal bone resorption caused by periodontitis.

**Fig. 2:**
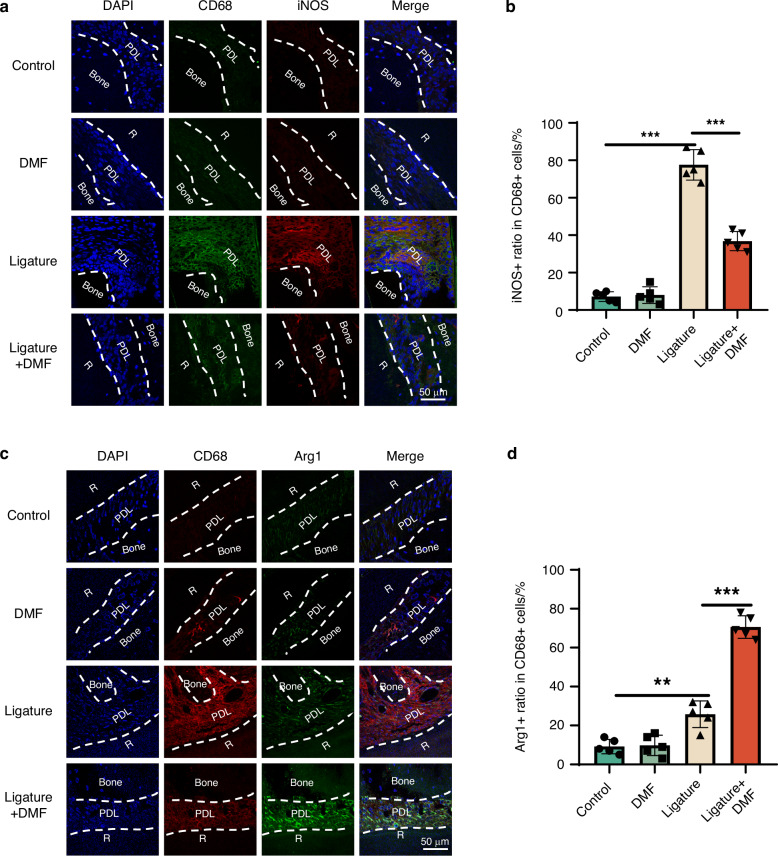
DMF modulates M1/M2 macrophage polarization in vivo. **a**, **c** Immunofluorescence staining of M1 polarization-related markers (iNOS/CD68) and M2 polarization-related markers (Arginase-1/CD68) and in macrophages across the periodontal region 14 days post-ligature. **b**, **d** Semi-quantitative analyses of iNOS+ and Arg1+ ratios in CD68+ cells. Data are presented as mean ± standard error of the mean. *P < 0.05, **P < 0.01, and ***P < 0.001 by one-way analysis of variance followed by Tukey's post hoc test. PDL periodontal ligament

Hematoxylin and eosin (H&E) and Tartrate-resistant acid phosphatase (TRAP) staining suggested that DMF may contribute to alveolar bone preservation and osteoclast formation inhibition in experimental periodontitis models (Fig. 1e–g). To further clarify the effect of DMF on macrophage differentiation in periodontal tissue, inducible nitric oxide synthase (iNOS) (M1 macrophages) and Arginase 1 (Arg1) (M2 macrophages) were co-stained with CD68. Compared with the control group, the ligation group had a higher proportion of M1- and M2-type macrophages, and using DMF effectively inhibited the generation of M1-type macrophages and promoted the differentiation into M2-type macrophages (Fig. 2a–d). Altogether, DMF effectively improved periodontal destruction and modulated M1/M2 macrophage polarization.

### DMF alters M1/M2 macrophage polarization in vitro

**Fig. 3:**
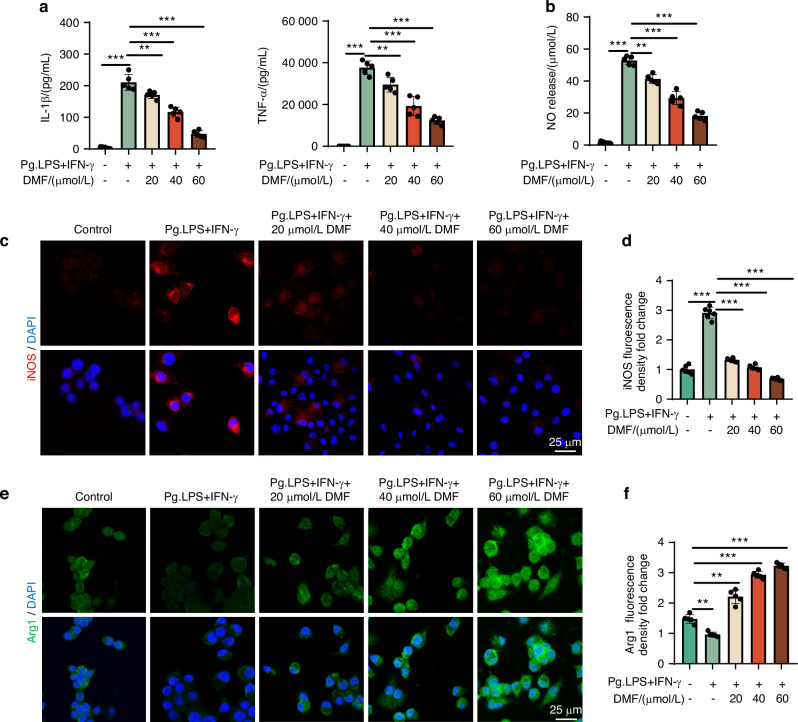
DMF alters M1/M2 macrophage polarization in vitro. **a** IL-1β and tumor necrosis factor-α protein concentration of RAW 264.7 cells tested using enzyme-linked immunosorbent assay. **b** NO concentration of RAW 264.7 cells tested using Griess assay. **c**, **e** Micrographs showing staining of iNOS, DAPI, and Arginase-1 using fluorescence microscopy. (Nucleus: blue, iNOS: red, Argianse-1: green.). **d**, **f** Semi-quantitative immunofluorescence analysis for iNOS and Arginase-1 in RAW 264.7 cells. Data are presented as the mean±standard error of the mean and are representative of ≥ 3 independent experiments. *P < 0.05, **P < 0.01, and ***P < 0.001 using *T*-test and one-way analysis of variance followed by Tukey's post hoc test

The balance between M1/M2 macrophages plays an important regulatory role in periodontitis development and recovery. Based on the results of the animal experiments, we speculated that DMF may alleviate periodontitis by regulating macrophage polarization. Macrophages were treated with DMF, and their phenotypes were evaluated to test this hypothesis. A high dose of DMF over 60 uM significantly inhibited cellular activity (Fig. S1c), and we chose various safe concentrations (20, 40, and 60 µmol/L) for cell proliferation in subsequent experiments. qRT-PCR revealed that DMF could effectively inhibit the transcription of iNOS and IL-1β while promoting the transcription of Arg1 and CD206 (Fig. S3a). Meanwhile, DMF could significantly reduce the release of IL-1β, TNF-α, and NO (Fig. 3a, b), indicating that DMF has excellent anti-inflammatory properties. To further clarify the effect of DMF on macrophage polarization at the protein level, we used immunofluorescence, western blotting (WB), and flow cytometry. Compared with the control group, iNOS expression in the Pg.LPS/IFN-γ + DMF group was significantly increased while the level of Arg1 was significantly decreased. When DMF was added, iNOS expression was significantly inhibited, whereas Arg1 expression was significantly increased (Fig. 3c–f; Fig. S3b-f), indicating that DMF could effectively regulate macrophage polarization. These results indicate that DMF can effectively inhibit macrophage inflammatory response and regulate macrophage polarization.

### DMF mitigates oxidative stress and mitochondrial dysfunction in vitro

Mitochondrial dysfunction and oxidative stress regulate macrophage polarization; therefore, we speculated that DMF may inhibit macrophage inflammation by protecting mitochondria and alleviating oxidative stress. Pg.LPS/IFN-γ upregulated mitochondrial ROS, decreased mitochondrial membrane potential (MMP), and reduced ATP levels, which were reversed by DMF (Fig. S4c–g). Moreover, DMF ameliorated cellular reactive oxygen species (ROS) (Fig. S4a, b) and increased malondialdehyde (MDA), superoxide dismutase (SOD), and Glutathione (GSH) levels (Fig. S4h–j). These results indicated that DMF may regulate macrophage polarization by alleviating mitochondrial dysfunction and oxidative stress. To test this hypothesis, we used MitoQ, a mitochondria-targeted antioxidant. The activated RAW 264.7 cells with MitoQ showed a reverse of mitochondrial dysfunction status and oxidative stress (Fig. S5a–j). Moreover, MitoQ effectively suppressed iNOS expression and elevated Arg1 expression on RAW264.7 cells (Fig. S5k, l). These findings suggest that DMF inhibits M1 macrophage polarization and promotes M2 macrophage polarization by mitigating oxidative stress and mitochondrial dysfunction.

### DMF attenuates oxidative damage and mitochondrial dysfunction by maintaining mitophagy in vivo and in vitro

**Fig. 4:**
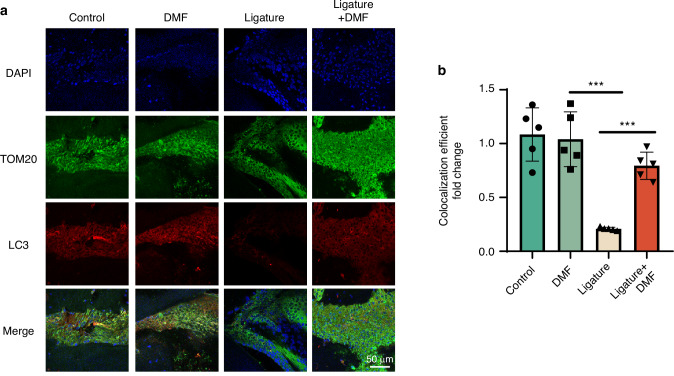
DMF induces mitophagy in vivo. **a** Immunofluorescence staining of LC3 and TOM20 in macrophages across the periodontal region 14 days post-ligature. **b** Co-localization of LC3 and TOM20 was analyzed using ImageJ. Data are shown as the mean±standard error of the mean and are representative of ≥ 3 independent experiments. *P < 0.05, **P < 0.01, and ***P < 0.001 using *T*-test and one-way analysis of variance followed by Tukey's post hoc test

**Fig. 5:**
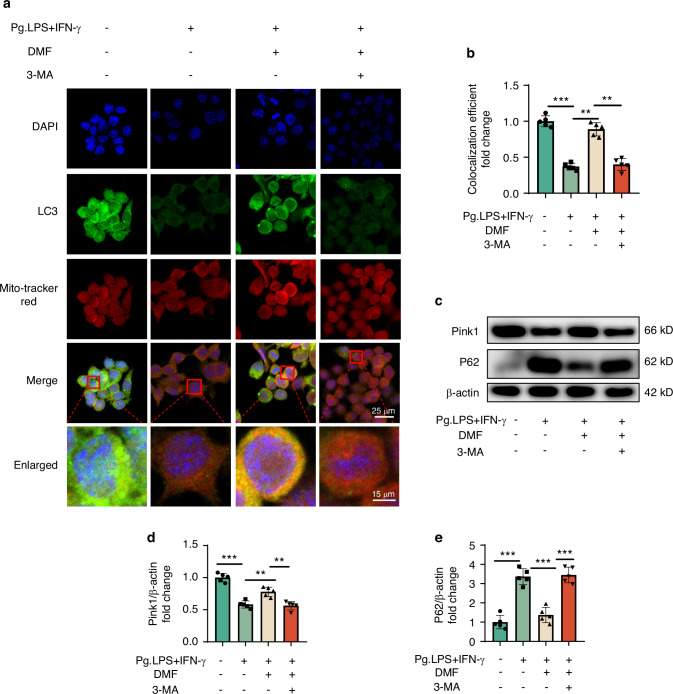
DMF induces mitophagy in vitro. **a** Micrographs showing staining of LC, DAPI, and Mitotracker using fluorescence microscopy. **b** Co-localization of LC3 and Mitotracker was analyzed using ImageJ. **c** Western blot band of Pink1 and P62 expressions in RAW 264.7 cells. **d**, **e** Pink1 and P62 levels relative to β-actin. Data are shown as the mean±standard error of the mean and are representative of ≥ 3 independent experiments. *P < 0.05, **P < 0.01, and ***P < 0.001 using T-test and one-way analysis of variance followed by Tukey's post hoc test

Mitophagy plays an important role in removing and protecting damaged mitochondria. Therefore, we suspected that DMF could protect the mitochondria and inhibit oxidative stress by maintaining mitophagy. We used the method of co-localization of LC3 with mitochondria to validate the level of mitophagy in mice periodontitis models and macrophages and found that DMF effectively promotes mitophagy in mice and RAW 264.7 cells (Fig. 4a–b; Fig. 5a–e;). We used 3-Methyladenine (3-MA), a potent mitophagy inhibitor, to verify whether mitophagy inhibition affects the efficacy of DMF. Adding 3-MA to RAW 264.7 cells hindered mitochondrial protection and the mitigation of oxidative damage by DMF (Fig. S6a–J). These results suggest that DMF protects the mitochondria and inhibits oxidative stress by enhancing mitophagy.

### DMF promotes mitophagy via TUFM in vivo and in vitro

**Fig. 6:**
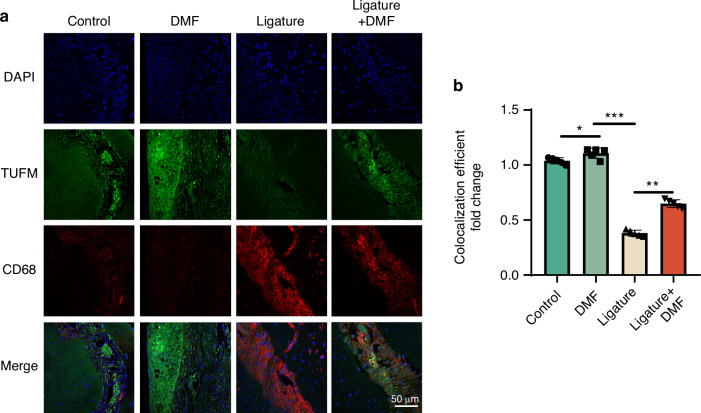
DMF promotes mitophagy via TUFM in vivo. **a** Immunofluorescence staining of TUFM and CD68 in macrophages across the periodontal region 14 days post-ligature. **b** Semi-quantitative analyses of TUFM+ ratio in CD68+ cells. Data are presented as the mean±standard error of the mean and are representative of ≥ 3 independent experiments. *P < 0.05, **P < 0.01, and ***P < 0.001 using T-test and one-way analysis of variance followed by Tukey's post hoc test

**Fig. 7:**
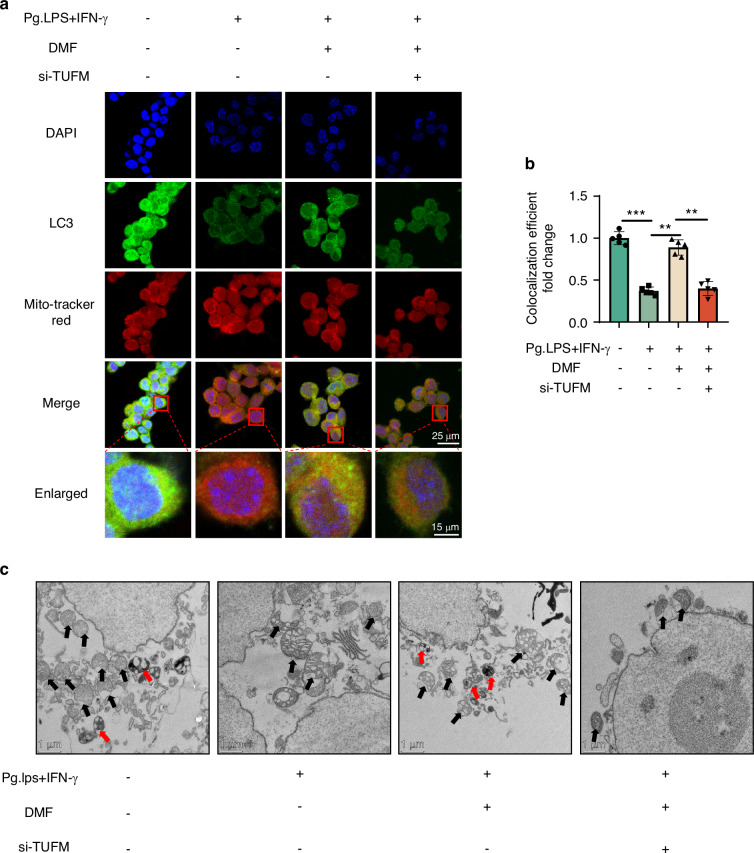
DMF promotes mitophagy via TUFM in vitro. **a** Micrographs showing staining of LC, DAPI, and Mitotracker using fluorescence microscopy. **b** The co-localization of LC3 and Mitotracker was analyzed using ImageJ. **c** Transmission electron microscopy images of mitochondrial and autophagosome. The red arrows indicate mitochondrion in the characteristic double-membrane autophagosomes, and the black arrows indicate mitochondria: scale bar, 500 nm. Data are presented as the mean±standard error of the mean and are representative of ≥ 3 independent experiments. *P < 0.05, **P < 0.01, and ***P < 0.001 using T-test and one-way analysis of variance followed by Tukey's post hoc test

Decreased TUFM levels, a recently identified key protein in regulating mitophagy, lead to decreased levels of mitophagy, mitochondrial dysfunction, and oxidative stress and exacerbate tissue inflammation. Given the important regulatory role of TUFM in mitochondrial homeostasis and redox balance and our previous experimental results, we speculated that DMF may exert a beneficial effect via TUFM. Staining showed that intracellular TUFM levels were significantly inhibited in periodontal tissues of the mouse model of periodontitis (Fig. 6a, b). Moreover, when macrophages were activated with Pg.LPS/IFN-γ, intracellular TUFM decreased significantly (Fig. S2a), while using DMF reversed the decrease in TUFM level (Fig. S7a, b), implying that the decreased TUFM level may be a mechanism for periodontitis pathogenesis. Previous experiments have demonstrated that DMF promotes mitophagy and simultaneously increases TUFM levels; therefore, we speculated that DMF may regulate mitochondrial autophagy through TUFM. We detected the level of mitophagy in macrophages using immunofluorescence and WB techniques and found that the DMF+Pg.LPS/IFN-γ group exhibited increased levels of mitophagy compared with the Pg.LPS/IFN-γ groups, such as an increase in Pink1 level and a decrease in P62, an increase in the co-localization coefficient between mitochondria and autophagosomes, and an increase in the number of mitochondria phagocytosed by autophagosomes under electron microscopy. After TUFM small interfering RNA (si-TUFM) transfection, the effects of DMF were eliminated (Fig. 7a–c; Fig. S7c–e). The above experimental results suggest that TUFM has a critical regulatory function in periodontitis development and that DMF promotes mitophagy by modulating TUFM levels.

### DMF attenuates oxidative damage and mitochondrial dysfunction via TUFM-mediated mitophagy in vitro

Given that previous studies have demonstrated that DMF can increase TUFM levels and alleviate mitochondrial dysfunction, we speculated that DMF may alleviate mitochondrial dysfunction and oxidative stress via TUFM. DMF effectively alleviated mitochondrial dysfunction caused by Pg.LPS/IFN-γ via TUFM (Fig. S8c–g). To investigate whether DMF alleviated Pg.LPS/IFN-γ-induced oxidative damage via TUFM, we used siRNA transfection to verify whether TUFM knockdown could block the antioxidant effect of DMF. Dichloro-dihydro-fluorescein diacetate (DCFH-DA) fluorescence intensity was significantly increased in activated macrophages, whereas adding DMF significantly attenuated the intracellular ROS level, and the mitochondrial ROS level was significantly increased after TUFM knockdown (Fig. S8a, b). MDA levels were significantly enhanced in the Pg.LPS/IFN-γ group, whereas it increased significantly after treatment with DMF. After the TUFM knockdown, the MDA level was significantly increased (Fig. S8h). The analysis of intracellular GSH level and SOD activity assay showed that Pg.LPS/ + IFN-γ significantly inhibited GSH levels and SOD activity. The GSH levels and SOD activity were restored significantly by DMF pretreatment. After TUFM knockdown, GSH levels and SOD activity were substantially reduced (Fig. S8i, j). These findings suggest that DMF may alleviate the oxidative damage and mitochondrial dysfunction induced by Pg.LPS/IFN-γ through TUFM.

### DMF inhibited TUFM degradation via the ubiquitin-proteasome pathway, not through the lysosome pathway

**Fig. 8:**
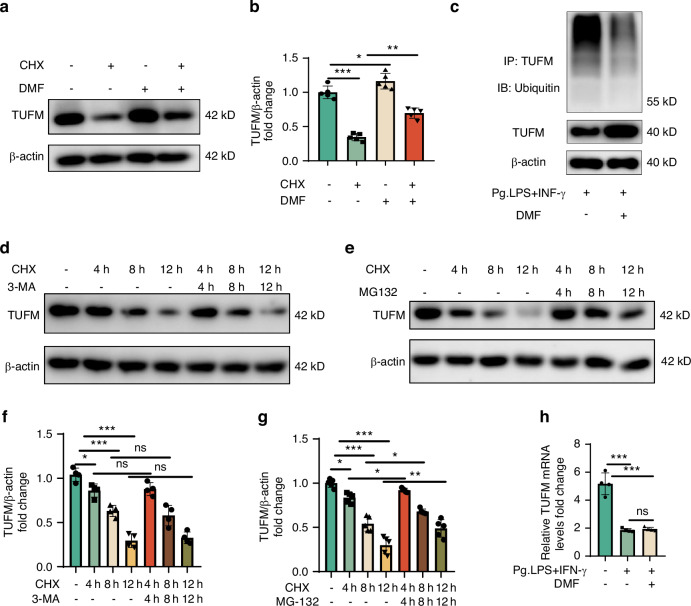
DMF inhibited TUFM degradation via the ubiquitin-proteasome pathway, not through the lysosome pathway. **a**, **d**, and **e** Western blot band of TUFM expression in RAW 264.7 cells. **b**, **f**, and **g** TUFM level relative to β-actin. **h** Real-time polymerase chain reaction analysis of the gene expression of TUFM. **c** Ubiquitination experiment of TUFM. Data are presented as the mean±standard error of the mean and are representative of ≥ 3 independent experiments. *P < 0.05, **P < 0.01, and ***P < 0.001 using T-test and one-way analysis of variance followed by Tukey's post hoc test

We have previously demonstrated that DMF increases TUFM levels and exerts protective effects through TUFM. Therefore, we sought to understand how DMF regulates TUFM. We first explored the regulation of the mRNA levels of TUFM by DMF. DMF did not restore the decreased TUFM transcript levels after stimulation (Fig. 8h). Therefore, we explored whether DMF increases TUFM levels by protecting against protein degradation. The results showed a significant reduction in TUFM levels after adding the protein synthesis inhibitor cycloheximide (CHX) for 12 h, and adding DMF significantly enhanced TUFM levels (Fig. 8a, b). The proteasome and lysosomal pathways are the two main pathways for intracellular protein degradation. Therefore, we explored which DMF pathway mainly relies on the regulation of TUFM protein expression. Adding the lysosomal inhibitor 3-MA did not alleviate the temporal gradient degradation level of TUFM, whereas adding proteasome inhibitor MG132 significantly alleviated the degradation of TUFM (Fig. 8d–g). Next, we implemented a ubiquitination experiment and found that TUFM up-regulation was accompanied by reduced ubiquitination with DMF (Fig. 8c). Therefore, we conclude that DMF protects TUFM from degradation by suppressing the ubiquitin-proteasome pathway.

### DMF modulates M1/M2 macrophage polarization via TUFM-mediated mitophagy in vitro

**Fig. 9:**
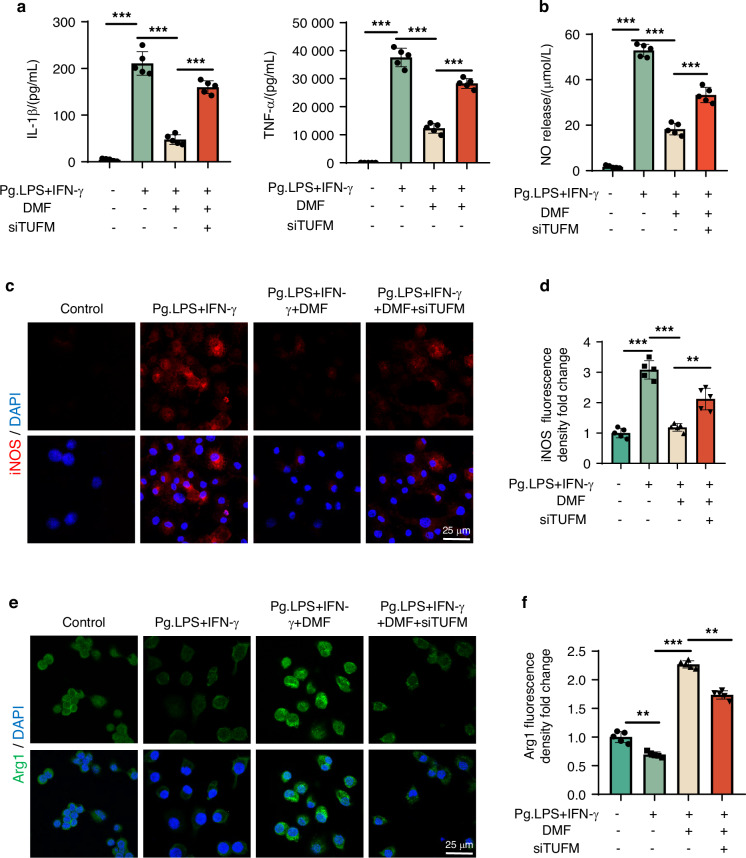
Dimethyl fumarate induces a shift in the polarization phenotype of macrophages in vitro. **a** IL-1β and TNF-α concentration of RAW 264.7 cells tested using enzyme-linked immunosorbent assay. **b** NO concentration of RAW 264.7 cells tested using Griess assay. **c**, **e** Micrographs showing staining of iNOS, DAPI, and arginase-1 using fluorescence microscopy. (Nucleus: blue, iNOS: red, Argianse-1: green.). **d**, **f** Semi-quantitative immunofluorescence analysis for iNOS and Arginase-1 in RAW 264.7 cells. Data are presented as the mean±standard error of the mean and are representative of ≥ 3 independent experiments. *P < 0.05, **P < 0.01, and ***P < 0.001 using T-test and one-way analysis of variance followed by Tukey's post hoc test

To clarify whether DMF regulates macrophage polarization after Pg.LPS/IFN-γ treatment via TUFM, we first tested the expression of M1 and M2 macrophage-related factors at the mRNA level. iNOS and IL-1β expressions at the mRNA level were significantly decreased in the LPS/IFN-γ + DMF group compared with the LPS/IFN-γ group, whereas the mRNA expression levels of Arg-1 and CD206 were significantly increased. After the si-TUFM intervention, iNOS and IL-1β expressions at the mRNA level increased significantly, whereas Arg1 and CD206 expressions at the mRNA level decreased significantly (Fig. S9a). We explored whether DMF affected macrophage polarization and phenotype through TUFM at the protein level using immunofluorescence and WB. Adding DMF potently reduced iNOS expression and promoted Arg1 expression. When TUFM was knocked down, iNOS levels increased significantly, whereas Arg1 levels decreased significantly (Fig. 9c–f; Fig. S9b–c). To further demonstrate that DMF protects against macrophage inflammation through TUFM, we examined IL-1β, TNF-α, and NO levels using ELISA and NO detection kits. IL-1β, TNF-α, and NO levels were significantly decreased in the Pg.LPS/IFN-γ + DMF group compared with the Pg.LPS/IFN-γ group, whereas TUFM knockdown elevated IL-1β and TNF-α inflammatory cytokines and NO release (Fig. 9a, b). The results suggest that DMF can promote the shift of the M1/M2 macrophage balance towards the M2 type and limit inflammatory factor release by regulating TUFM.

## Discussion

**Fig. 10:**
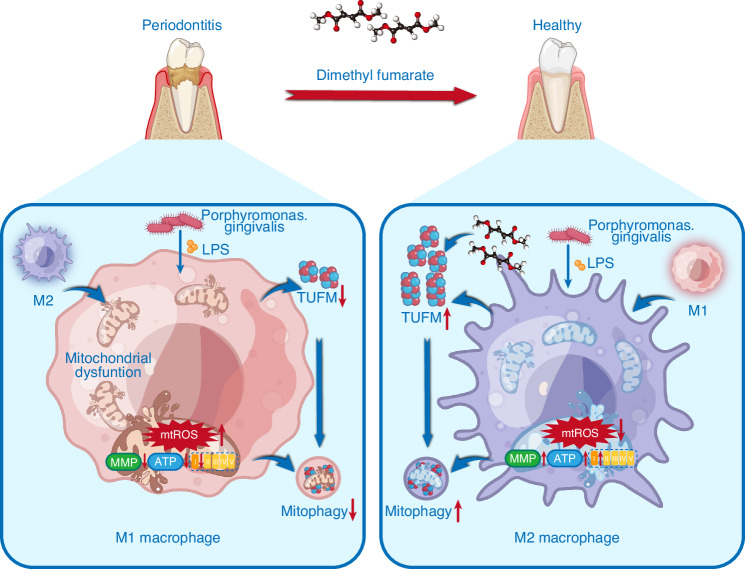
Schematic diagram for the beneficial effects of dimethyl fumarate against periodontitis through the regulation of TUFM dependent Mitophagy

Periodontitis is a common immune-mediated inflammatory disease leading to tooth loss. Given that the current treatment effects are unsatisfactory, studies have focused on regulating immunity to prevent or treat periodontitis.^1,2,5^ This study demonstrated, for the first time, that DMF, an antioxidant and immunomodulatory agent, ameliorated periodontitis by modulating macrophage polarization. Mechanistic studies indicated that DMF repressed macrophage mitochondrial dysfunction and oxidative stress via TUFM-mediated mitophagy and protected TUFM from degradation via the proteasome pathway, not the lysosome pathway (Fig. 10). Overall, TUFM may be a new target for preventing periodontitis and provides a scientific basis for applying DMF in treating periodontitis.

Numerous studies have shown that macrophages play a major role in regulating tissue homeostasis and the inflammatory response.^7,40^ Since M0 macrophage polarization to the M1 macrophage is primarily induced by Th1 factors such as IFN-γ and LPS released by bacteria in periodontal tissue,^6^ we chose Pg.LPS/IFN-γ as a stimulation for activation of macrophage. In this study, Pg.LPS/IFN-γ groups displayed increased and decreased proportion of M1 and M2 macrophages, respectively, compared with the control groups at protein and mRNA levels. Moreover, in a mouse periodontitis model, the proportion of macrophage subsets in periodontal tissues also showed significant changes. However, treatment with DMF effectively reversed these effects and consequently inhibited alveolar bone resorption and the release of inflammatory factors, suggesting that DMF alleviates periodontitis progression by modulating macrophage polarization. Previous studies have shown that DMF can weaken inflammatory microenvironment and promote the macrophages polarization to M2 by Nrf2 activation and an autophagy-dependent pathway.^36,42–44^ Interestingly, monomethyl fumarate, a metabolite of DMF, has similar effects on osteoarthritis by preventing and augmenting M1 and M2 macrophage polarizations, respectively,^42^ suggesting the protective role of the methyl ester of fumaric acid in inflammatory diseases.

Numerous studies have shown that mitochondrial dysfunction is an important mechanism for the imbalance in macrophage polarization and difficulty in converting M1-like to M2-like macrophages.^9,45^ Therefore, alleviating mitochondrial dysfunction is an important means of regulating macrophage polarization. Macrophages in Pg.LPS/IFN-γ groups exhibited mitochondrial dysfunction compared with the control groups, whereas the consequent DMF treatment effectively reversed it. Mitochondria are the main source of ROS and the target of oxidative damage. Several studies have shown that excessive ROS generation is an important inducer of M1 macrophage polarization, whereas the effective removal of ROS can reduce activated macrophages.^17,46,47^ We found that DMF effectively decreased ROS production (MDA) and increased antioxidant enzyme (GSH and SOD) production after Pg.LPS/IFN-γ treatment. Previous literature showed that DMF promotes NRF2 translocation into the nucleus via KEAP1 succination and dissociation of NRF2 and KEAP1, exerting antioxidant effects.^30,48^ Given the above findings, we speculated that DMF modulates macrophage polarization after stimulation with Pg.LPS/IFN-γ by protecting mitochondrial function and ameliorating oxidative stress. We used MitoQ, a mitochondrial target antioxidant, to test the hypothesis. MitoQ effectively inhibited and promoted M1 and M2 macrophage polarizations, respectively, which is consistent with the results of previous studies and in line with our hypothesis. A recent study showed that MnSOD knockdown, a mitochondrial antioxidant defense, inhibited the polarization and infiltration of M2 macrophages and enhanced M1 macrophage phagocytic,^49^ further validating the key regulatory role of mitochondrial homeostasis in macrophage polarization. In addition, recent reports suggests that metabolism plays an important role in the transition of the polarization phenotype of macrophages. M1 macrophages often undergo glycolysis to obtain energy, while M2 macrophages select oxidative phosphorylation and FAO.^50,51^ On the contrary, when macrophages undergo metabolic reprogramming, they will also transform into corresponding phenotypes.^50,52^ A recent study found that DMF can inhibit glycolysis by succinating and inactivating GADPH in activated myeloid and lymphoid cells, which mediates its anti-inflammatory effects,^35^ implying that the regulation of metabolic pathways is one of the important foundations of DMF in the treatment of periodontitis.

Mitophagy, a protective mechanism that selectively degrades damaged mitochondria, regulates the quality of mitochondria and redox balance.^19,53^ Nowadays, the mainstream view in academia is that stimulation with LPS/IFN-γ decreases the level of mitophagy in macrophages.^20,21^ When mitochondrial dysfunction occurs with mitophagy dysfunction, macrophages will inevitably polarize M1 macrophage and have difficulty switching to M2 macrophage.^9,54^ However, the role of mitophagy in macrophage polarization remains controversial. Ling et al. found that mitophagy level was significantly decreased in macrophages exposed to LPS.^55^ The concentration difference of LPS may contribute to the above contradictory results since Lin et al. used a lower LPS concentration (10 ng/mL) to activate macrophages compared with other studies, including ours (1 µg/mL). We showed that mitophagy levels in activated macrophages and periodontitis mouse models were significantly decreased compared with those in the control groups, implying that mitophagy is an important mechanism for periodontitis development. Meanwhile, DMF increased mitophagy levels and alleviated mitochondrial dysfunction and oxidative damage after stimulation with Pg.LPS/IFN-γ and 3-MA treatment, a mitophagy inhibitor, counteracted this protective effect, suggesting that DMF mitigates mitochondrial dysfunction and oxidative stress by enhancing mitophagy. Poojitha et al. showed that DMF enhances mitophagy, contributing to the prevention and treatment of Parkinson’s disease via the NRF2/BNIP3/PINK1 pathway.^34^ Pink1 knockout mice exhibited lower BV/TV combined with trabecular thickness and more osteoclasts than wild-type mice upon ligature placement.^56^ Therefore, maintaining mitochondrial homeostasis by promoting mitophagy may be an important therapeutic strategy for preventing and treating periodontitis.

TUFM, encoded by the mitochondrial gene, is a highly conserved GTPase that is part of the mitochondrial protein translation machinery.^26,27^ Previous studies have shown that TUFM downregulation affects the mitochondrial respiratory chain, leading to elevated ROS levels in several cell lines. TUFM overexpression partially rescues the defective assembly of the respiratory chain caused by tRNA mutations. In addition, TUFM is involved in oxidative stress and inflammation via mitochondrial oxidative phosphorylation and quality control.^27–29^ Because of the critical role of TUFM in maintaining normal cellular functions, we suggest that TUFM may be a novel therapeutic target for periodontitis. We found, for the first time, that adding Pg.LPS/IFN-γ resulted in a significant decrease in the intracellular TUFM level in macrophages, which had similar results in the mouse periodontitis model, indicating that the decrease of TUFM is an important mechanism in periodontitis development. We found that using DMF reversed the decline in TUFM levels, implying that DMF exerts protective effects by increasing TUFM levels. TUFM is a mitochondrial membrane protein involved in maintaining mitochondrial homeostasis. A recent study has shown that TUFM regulates mitophagy to alleviate the progression of nonalcoholic steatohepatitis by removing damaged mitochondria.^29^ Given the regulation and function of TUFM and the biological role of DMF, we suggest that DMF alleviates mitochondrial dysfunction and oxidative damage through TUFM-mediated mitophagy. We found that DMF increased mitophagy via TUFM, which is consistent with previous findings. Importantly, we showed that the protective effect of DMF on mitochondrial and redox balance was eliminated after adding si-TUFM, verifying our conjecture. TUFM can regulating the release of mtDNA and form a complex with the mitochondrial protein NLRX1, which reduce the inflammatory response.^27,57^ In the future, we will explore whether DMF can regulate macrophage polarization through the release of mtDNA regulated by TUFM or the formation of TUFM-NLRX1 complex.

Considering that DMF can significantly increase TUFM levels, we first measured the mRNA levels of TUFM to explore how DMF regulates TUFM expression. qRT-PCR showed that DMF was not conducive to the increased mRNA levels of TUFM and did not explain the regulatory mechanism of DMF on TUFM; therefore, we speculated that DMF protects TUFM from degradation. Only MG132, not 3-MA, alleviated the significant decrease in TUFM levels after adding CHX. Therefore, we suggest that DMF regulates TUFM levels by inhibiting the proteasomal pathway but not the lysosomal pathway. Chang-Yong et al. found that when the synthesis of TUFM is completed, it is transferred to the outer mitochondrial membrane and quickly ubiquitinated by Ub E3 ligase for proteasome-mediated degradation,^58^ which strongly suggests that DMF may maintain TUFM levels by inhibiting the proteasome degradation pathway. Based on the above discussion, we speculate that DMF protects TUFM from degradation by inhibiting its ubiquitination; however, the exact mechanism needs to be verified.

Although we identified the therapeutic mechanism of DMF in periodontitis and revealed a novel role for TUFM in macrophage polarization via mitophagy, this study had some limitations. First, we chose RAW264.7 cells instead of Bone-marrow-derived macrophage (BMDM) cells to establish an in vitro cell model. Given some drawbacks of cell lines, primary BMDMs can better validate our hypothesis than research models closer to the in vivo environment. Second, the beneficial effects of DMF on periodontitis via TUFM were demonstrated only in vitro using siRNA or not TUFM-knockout mice. In the future, the underlying regulatory mechanism of DMF on TUFM needs to be further explored by using TUFM knockout mice. Finally, in view of the side effects caused by oral administration of DMF, we will prepare novel hydrogel loading DMF to reduce the side effects in the future.

## Methods

### Animal experimental periodontitis models

The Animal Ethics Committee of Wenzhou Medical University (wydw2023-0278) approved the experiments. Six-week-old C57BL/six male mice were randomly divided into six groups: C: no treatment; DMF: Mice treated with DMF; P: Experimentally induced periodontitis; P + DMF 50: periodontitis mice treated with 50 mg/kg DMF; P + DMF 100: periodontitis mice treated with 100 mg /Kg DMF; and P + DMF 150: periodontitis mice treated with 150 mg/kg DMF. DMF was added to sodium carboxymethyl cellulose and stored at 4 °C for subsequent experiments. The DMF and DMF + P groups were administered DMF using gavage once daily. The 6-week-old mice in the DMF and DMF + P groups were pretreated with DMF for 1 week. One week later, we induced periodontitis using a 5-0 silk thread for two weeks, and the DMF and DMF + P groups were administered with DMF for 2 weeks. Generally, 7-week-old mice were first anesthetized and fixed using a fixation device. The silk thread was clamped using ophthalmic forceps and placed at the neck of the second molar. The silk thread was checked every two days. If the silk thread became loose, it was ligated on the same day.

### Analysis of alveolar bone loss

Stereomicroscopy was used to detect the distance between the cemento-enamel junction (CEJ) and alveolar crest (AC). The region of interest (ROI) was an rregular bone body between the first and second molars. Bone mineral density (BMD) and bone volume over total volume (BV/TV) were further analyzed using the SkyScan 1276 software.

The soft tissue of each alveolar bone was removed and photographed using a stereomicroscope. Images were used to measure the distance between the CEJ and AC. Micro-CT was used to obtain X-ray images of the alveolar bone. The region between the first and second molar roots was selected as the ROI. SkyScan software was used to analyze the images and obtain BMD and BV/TV data. Red boxes were used to represent the regions of interest.

### Histological analysis

The processed samples were fixed in paraformaldehyde (PFA) at 4 °C for 1 day and decalcified with EDTA for 24 days. The samples were then embedded in liquid paraffin. The embedded samples were sectioned into 5 μm thickness for subsequent staining. Inflammatory infiltration was evaluated using H&E staining, and the number of osteoclasts was measured using TRAP staining.

### Histological immunostaining

The samples were washed thrice with phosphate-buffered saline (PBS) for 3 min each. We put the corresponding fluorescent secondary antibody onto the tissue and incubated it in a 37 °C constant temperature incubator for 1 h in the dark. After incubating, the samples were washed thrice with PBS for 3 min each. We put the sealing agent containing DAPI onto the samples and sealed it with a glass slide. Fluorescent images were captured using laser scanning confocal microscopy (LSCM).

### Cell culture and activation

RAW 264.7 cells were maintained in Dulbecco’s modified eagle medium containing 10% fetal bovine serum (Gibco, USA) and 0.1% penicillin-streptomycin solution at 37 °C with 5% CO_2_. For medical treatment, macrophage was pretreated using DMF (20, 40, and 60 μmol/L) or for 2 h after reaching 70% confluence. For macrophage activation, cells were stimulated by using Porphyromonas gingivalis lipopolysaccharide (Pg.LPS) (1 μg/mL) and interferon-γ (IFN-γ) (20 ng/mL) for 22 h.

### Quantitative real-time polymerase chain reaction (qRT-PCR)

After removing the culture medium, 1 mL of TRIzol was directly added to the culture plate to lyse the cells, and total RNA was extracted using TRIzol. cDNA was synthesized using reverse transcriptase (Takara Biotechnology, Shiga, Japan). A SYBR Green PCR Master Mix Kit (Takara) was used for qRT-PCR. The exported data was analyzed using the ΔΔCT method and normalized to β-Actin. The primer sequence is shown in Table S1.

### Western blot

The total protein of RAW 564.7 cells was extracted using radioimmunoprecipitation assay lysis solution, and a bicinchoninic acid assay Kit (Beyotime) was used to measure the protein concentration of the sample. One-third of the volume of the 4× loading buffer was added to the protein lysis solution, and the sample was boiled for 10 min. Each sample was added to a lane of a sodium dodecyl sulfate-polyacrylamide gel. After electrophoresis, the proteins were transferred onto the polyvinylidene fluoride membrane (Thermo Fisher Scientific). The membrane was blocked with 5% milk at room temperature and incubated overnight with primary antibody against inducible nitric oxide synthase (iNOS) (1:1 000, Thermo), Arginase-1 (1:5 000, protein tech), β-actin (1:8 000, Abcam) LC3 (1:1 000, MBL), Parkin (1:1 000, CST), P62 (1:1 000, Santa), Pink1 (1:1 000, Santa), TUFM (1:4 000, abcam) overnight at 4 °C. The following day, the membranes were incubated with the corresponding secondary antibody (Beyotime) for 1.5 h at room temperature. Finally, a chemiluminescence kit (Solarbio) was used to detect target protein bands.

### Detecting oxidative stress

Macrophages were maintained in 24-well plates at a density of 1 × 10^5^ cells per well for further analysis. After being stimulated and treated with DMF, the cells were incubated with DCFH-DA (1:1 000, Beyotime) for 30 min at 37 °C and washed thrice with PBS. Fluorescence images were obtained using a fluorescence microscope (Zeiss). Lipid peroxidation levels were measured using a malondialdehyde (MDA) detection kit (Beyotime). Glutathione (GSH) levels were measured using a GSH Assay Kit. The superoxide dismutase (SOD) levels were tested by using a total SOD assay kit (Beyotime).

### Detecting mitochondrial dysfunction

Macrophages were seeded into 24-well plates at a density of 1 × 10^5^ cells per well for 24 h. The cells were stimulated by Pg.LPS + INF-γ and treated with DMF for 24 h. After treatment, the cells were incubated with TMRM (1 μmol/L, Thermo) and Mitogreen probes (1 μmol/L, Thermo) for detecting mitochondrial membrane potential (MMP) and Mitosox probes (1 μmol/L, Thermo) + Mitogreen probes (1 μmol/L, Thermo) for detecting mitochondrial ROS level. ATP levels were measured using an ATP assay kit (Beyotime).

### Enzyme-linked immunosorbent assay (ELISA) and NO measurement

Macrophages were seeded into 96-well plates at a density of 1.5 × 10^5^ cells per well for 24 h. Following stimulation and treatment, the cell supernatant was collected to detect the concentration of tumor necrosis factor-α (TNF)-α and interleukin-1β (IL-1β) using an ELISA kit. NO expression was detected using the Griess reagent (Beyotime).

### Immunofluorescence staining

Macrophages were maintained on slides for subsequent experiments. Following stimulation and treatment, the cell supernatant was removed, and PBS containing 0.5 μmol/L of MitoTracker Red (500 nmol/L, Thermo) was added to each well and incubated at room temperature for 10 min. The samples were washed thrice with PBS for 3 min each and fixed with 4% paraformaldehyde (PFA). After fixation, the cells were washed thrice with PBS for 3 min each. The cells were permeabilized with PBS containing 0.1% Triton X-100 for 10 min and washed thrice with PBS for 3 min each. The slides were blocked with 5% bovine serum albumin (Sigma) at room temperature for 45 min. We added PBS containing antibody anti-iNOS (1:200, Thermo), anti-Arginase-1 (1:200, Thermo), and anti-LC3 (1:200, MBL) dropwise to each sample and overnight at 4 °C. On the second day, we washed the samples thrice with PBS for 3 min each, and we added the corresponding fluorescent secondary antibody and incubated at 37 °C for 1 h. After incubating, the slides were washed thrice with PBS for 3 min each. The slides were sequentially spotted with a mounting medium containing DAPI and covered with a coverslip. Immunofluorescence images were captured using LSCM.

### Transmission electron microscopy

RAW 264.7 cells were isolated in an Eppendorf tube and centrifuged to remove the supernatant. Thereafter, they were immediately fixed in an electron microscope fixative and stored in a 4 °C refrigerator. The solution was washed thrice with PBS for approximately 10 min each to wash off the residual glutaraldehyde. We added 1–2 drops of 1% osmic acid to the tissue sample and allowed it to stand for 1 h at room temperature. We washed twice with the buffer for approximately 10 min each. An appropriate amount of uranium acetate was added to fully infiltrate the tissue block, which was left to stand at room temperature for staining for 1–2 h. The tissue blocks were successively immersed in 30%, 50%, 70%, 80%, 90%, and 100% (twice) acetone solutions for gradient dehydration, respectively, and each concentration was applied for approximately 10 min. The cell pellets were immersed in an embedding solution. The samples were cut into 70–90 nm sections, which were observed under an h-7500 transmission electron microscope at 80 kV.

### Flow cytometry

Macrophages were cultured in 6-well plates at a density of 1 × 10^6^ for 24 h. After pretreatment and stimulation, the cells were collected, fixed with 4% PFA for 10 min, and rinsed thrice with PBS. Cells were blocked by CD16/32 (1:200, Thermo) and subsequently incubated with iNOS (1:1 000, Thermo) and Arg-1 (1:5 000, protein tech) at 4 °C for 1 h. The cells were rinsed with PBS and assessed using flow cytometry. The data were analyzed using FlowJo v10 software.

### Statistical analysis

Data are presented as mean ± standard deviation and analyzed using GraphPad Prism software. All data were obtained from more than three independent analyses of experimental results. A one-way analysis of variance was used to compare more than two groups. *P < 0.05, **P < 0.01, and ***P < 0.001 were used to indicate significant differences.

## Supplementary information

Supplemental material

## Data Availability

The data that support the findings of this study are available on request from the corresponding author. The data are not publicly available due to privacy or ethical restrictions.
