# Supplementary material for: Dimethyl fumarate modulates M1/M2 macrophage polarization to ameliorate periodontal destruction by increasing TUFM-mediated mitophagy
Source: Int J Oral Sci. 2025 Apr 17;17:32. doi: 10.1038/s41368-025-00360-0 (PMC12006468; doi:10.1038/s41368-025-00360-0)
Supplement: Supplementary file 1 — Supplemental material [file 41368_2025_360_MOESM1_ESM.doc]

**Supplementary material**

**Dimethyl fumarate modulates M1/M2 macrophage polarization to ameliorate periodontal destruction by increasing TUFM-mediated mitophagy**

Liang Chen ^1#^, Pengxiao Hu ^1#^, XinHua Hong ^1#^, Bin Li ^1^, Yifan Ping ^1^, ShuoMin Chen ^1,2^, Tianle Jiang ^1^, Haofu Jiang ^1^, Yixin Mao ^1,2^, Yang Chen^1,2^, Zhongchen Song^5,6,7^, Zhou Ye^8*^, Xiaoyu Sun^1,3*^, ShuFan Zhao ^1,4*^, ShengBin Huang ^1,2*^

#These authors made equal contributions to this work.

*Corresponding authors: ShuFan Zhao, E-mail: [sf.zhao@wmu.edu.cn;](mailto:sf.zhao@wmu.edu.cn;) ShengBin Huang, E-mail: huangsb003@wmu.edu.cn.

**Affiliation(s)**

1. Institute of Stomatology, School and Hos pital of Stomatology, Wenzhou Medical University, Wenzhou, China
2. Department of Prosthodontics, School and Hospital of Stomatology, Wenzhou Medical University, Wenzhou, China
3. Department of Periodontology, School and Hospital of Stomatology, Wenzhou Medical University, Wenzhou, China
4. Department of Oral Maxillofacial Surgery, School and Hospital of Stomatology, Wenzhou Medical University, Wenzhou, China
5. Department of Periodontology, Shanghai Ninth People’s Hospital, Shanghai Jiao Tong University School of Medicine, Shanghai, China
6. College of Stomatology, Shanghai Jiao Tong University, Shanghai, China
7. National Center for Stomatology, National Clinical Research Center for Oral Diseases, Shanghai Key Laboratory of Stomatology, Shanghai, China
8. Applied Oral Sciences and Community Dental Care, Faculty of Dentistry, University of Hong Kong, Hong Kong, China.

**
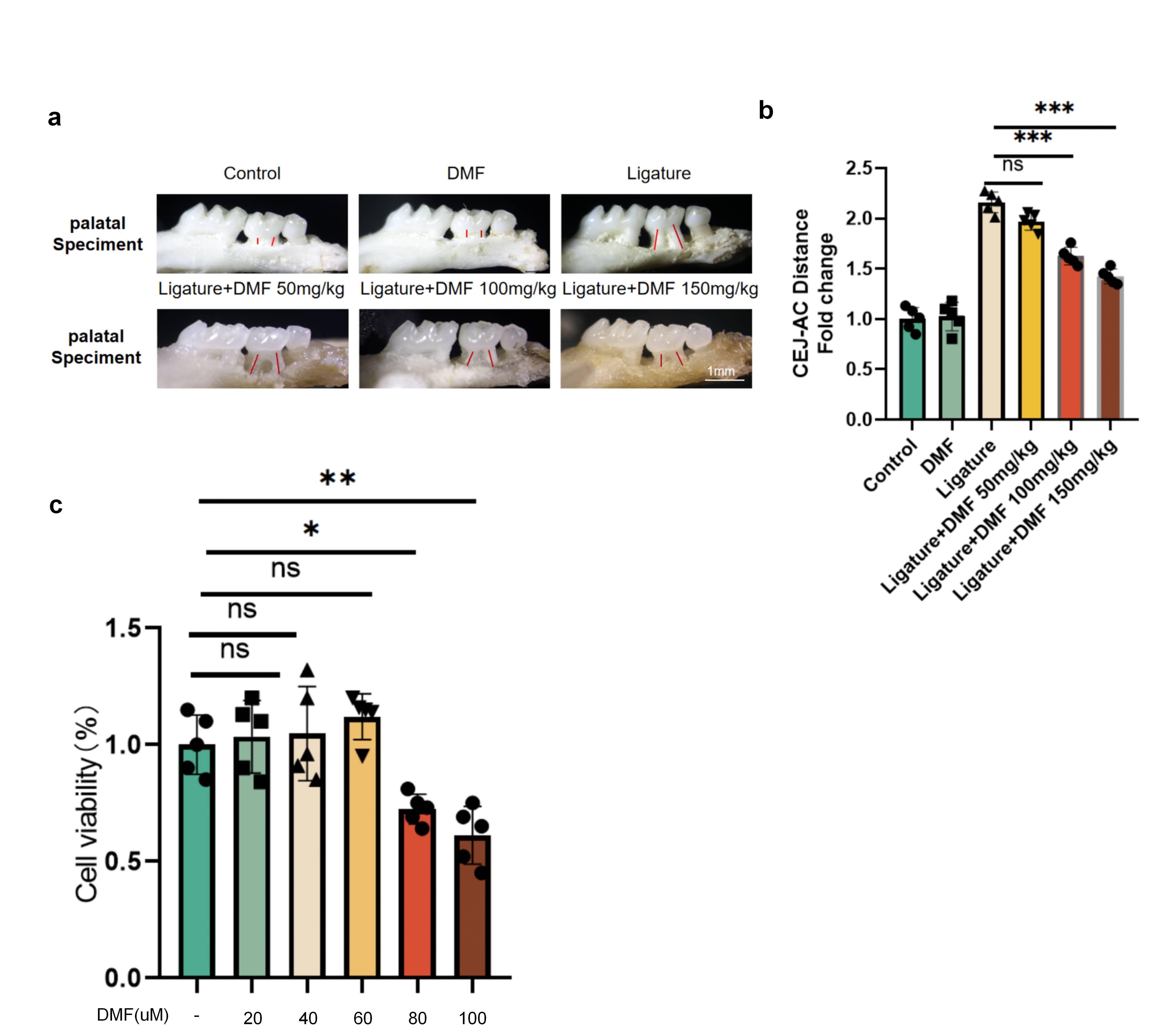
**

Figure S1. **a** Macroscopic aspects of the maxilla in mice from Control, DMF, Ligature, Ligature+DMF 50 mg/kg, Ligature+DMF 100 mg/kg and Ligature+DMF 150 mg/kg groups with volume microscope and 3D reconstruction with micro-computed tomography. The red lines showed the distance from the elemental junction (ACJ) to the AC. **b** Quantitative analysis of ACJ-AC distance (normalized to the Control group). **c** CCK8 assay to quantity cell viabilities with DMF. Data are presented as the mean±standard error of the mean. *p < 0.05, **p < 0.01, and ***p < 0.001 using one-way analysis of variance followed by Tukey's post hoc test.

AC, alveolar crest; ACJ, amelocemental junction; DMF, dimethyl fumarate


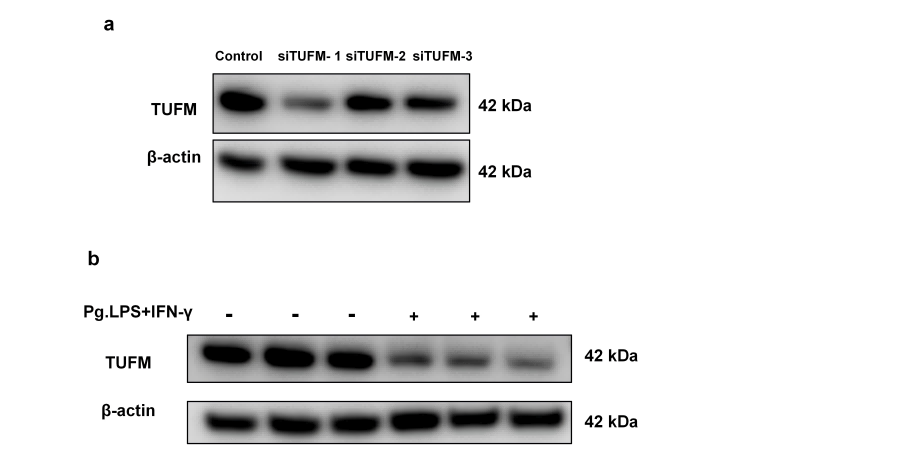


Figure S2. **a** Transfection effects of TUFM siRNA analyzed using Western blot band. **b** Western blot band of TUFM expression in RAW 264.7 cells stimulated by Pg.LPS/IFN-γ or not.


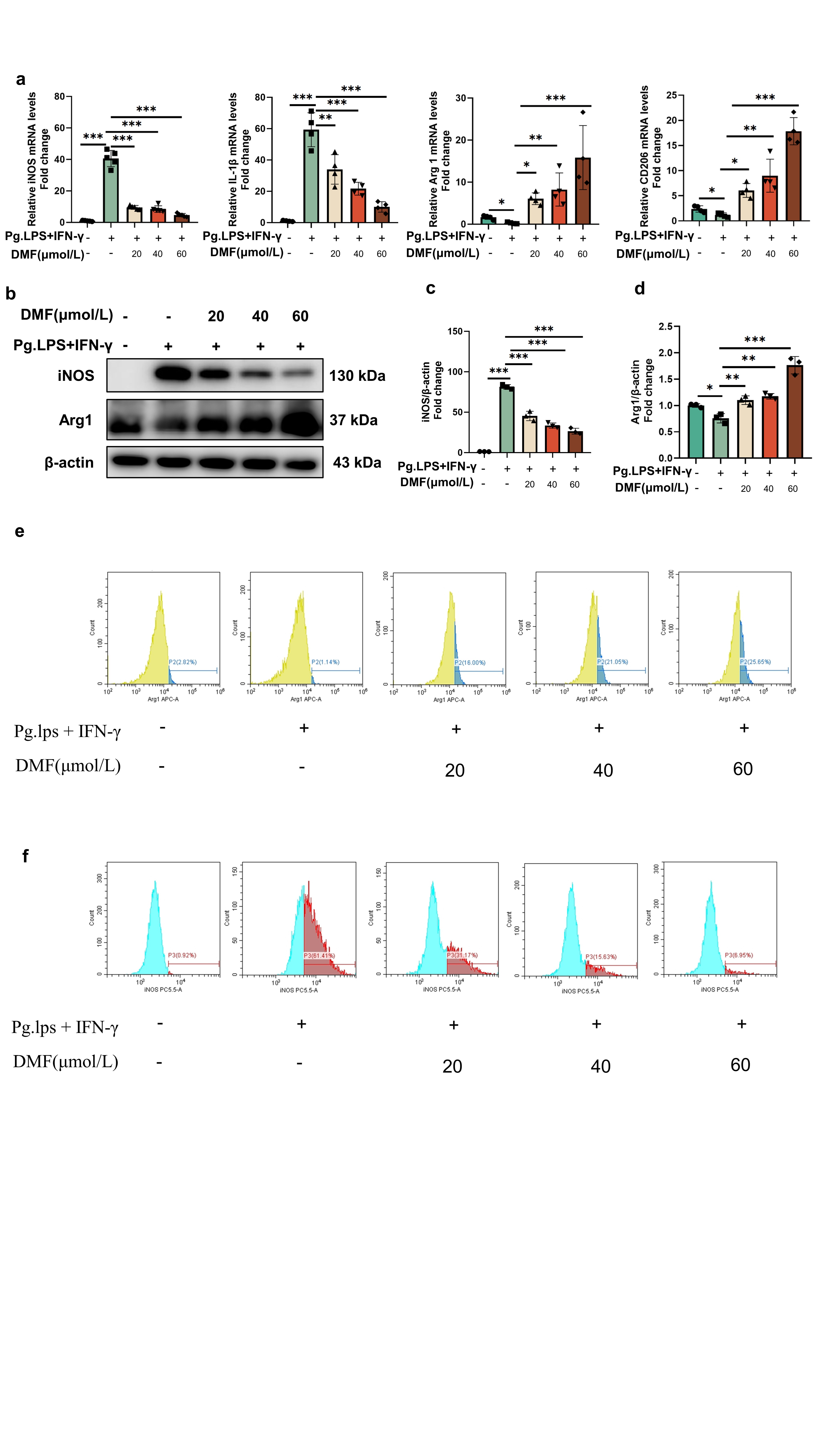


Figure S3. **a** Real-time polymerase chain reaction analysis of the gene expression of the M1-related iNOS and IL-1β and the M2-related Arg1 and CD206. **b** Western blot band of iNOS and Argianse-1 expression in RAW 264.7 cells. **c, d** Level of iNOS and Argianse-1 relative to β-actin. **e, f** Flow cytometry analysis of the M1-related marker iNOS and the M2-related marker Arg-1. Data are presented as the mean±standard error of the mean and are representative of ≥3 independent experiments. *p < 0.05, **p < 0.01, and ***p < 0.001 using T-test and one-way analysis of variance followed by Tukey's post hoc test.


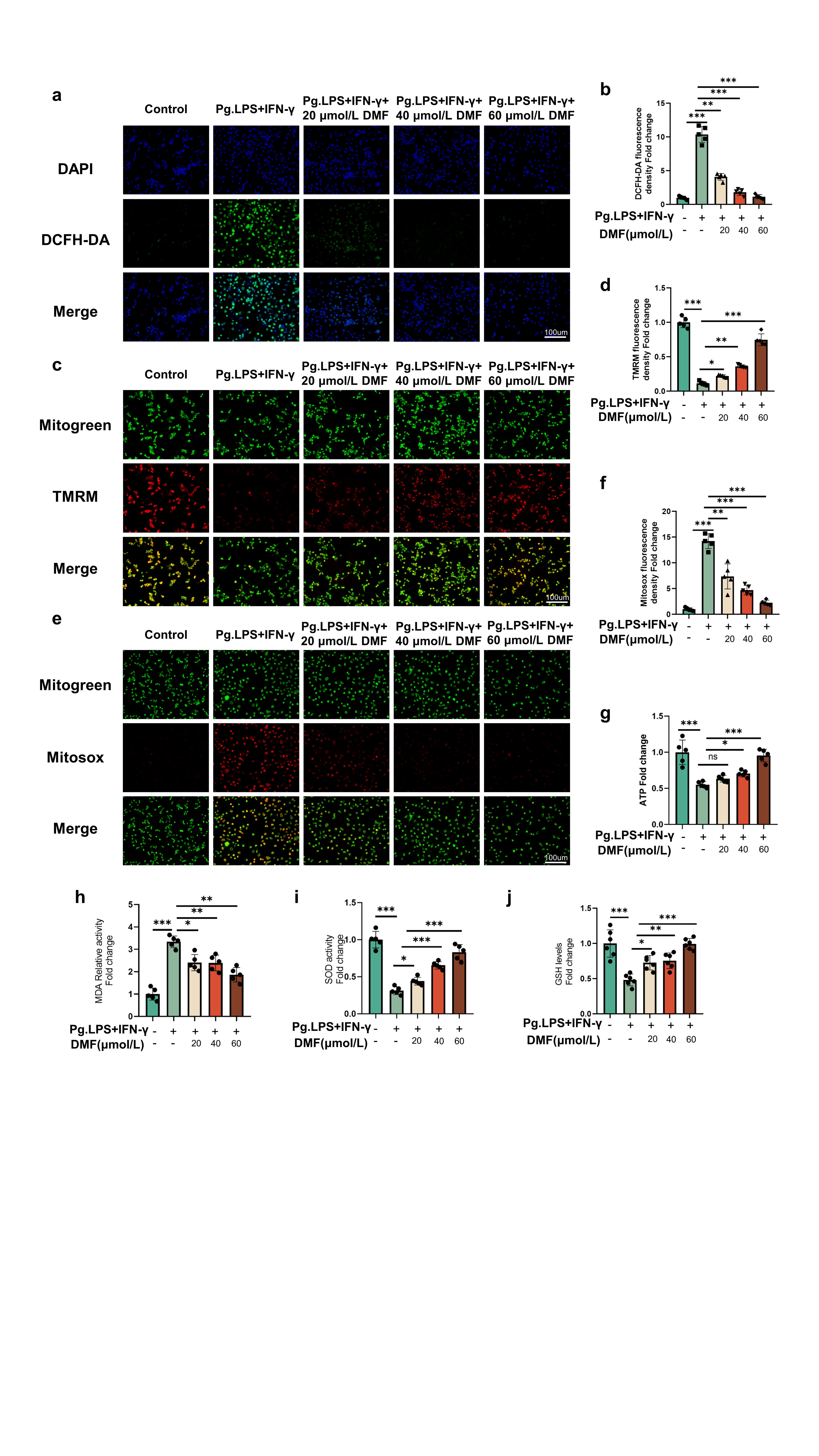


Figure S4. **a**Representative images of DCFH-DA staining (scale bar = 100 um). **b**Semi-quantitative analysis of immunofluorescence for DCFH-DA staining. **c** Representative images of TRM staining (scale bar = 100 um). **d** Semi-quantitative analysis of immunofluorescence for TMRM staining. **e**Representative images of Mitosox staining (scale bar = 100 um). **f**Semi-quantitative analysis of immunofluorescence for Mitosox staining. **g** The ATP content of RAW 264.7 cells. **h** The MDA content of RAW 264.7 cells. **i**The SOD content of RAW 264.7 cells. **j** The GSH content of RAW 264.7 cells. Data are presented as the mean±standard error of the mean and are representative of ≥3 independent experiments. *p < 0.05, **p < 0.01, and ***p < 0.001 using T-test and one-way analysis of variance followed by Tukey's post hoc test.


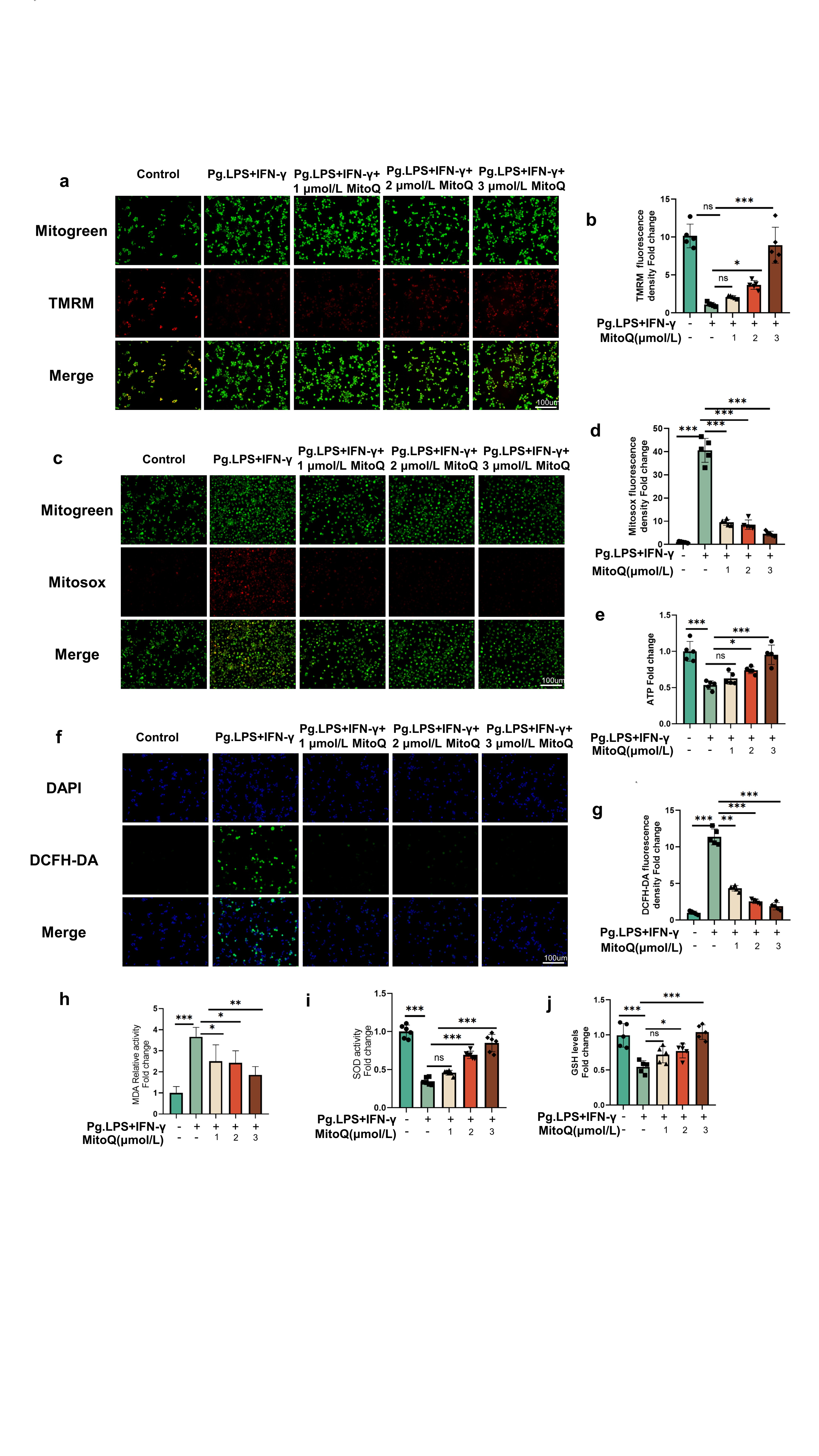


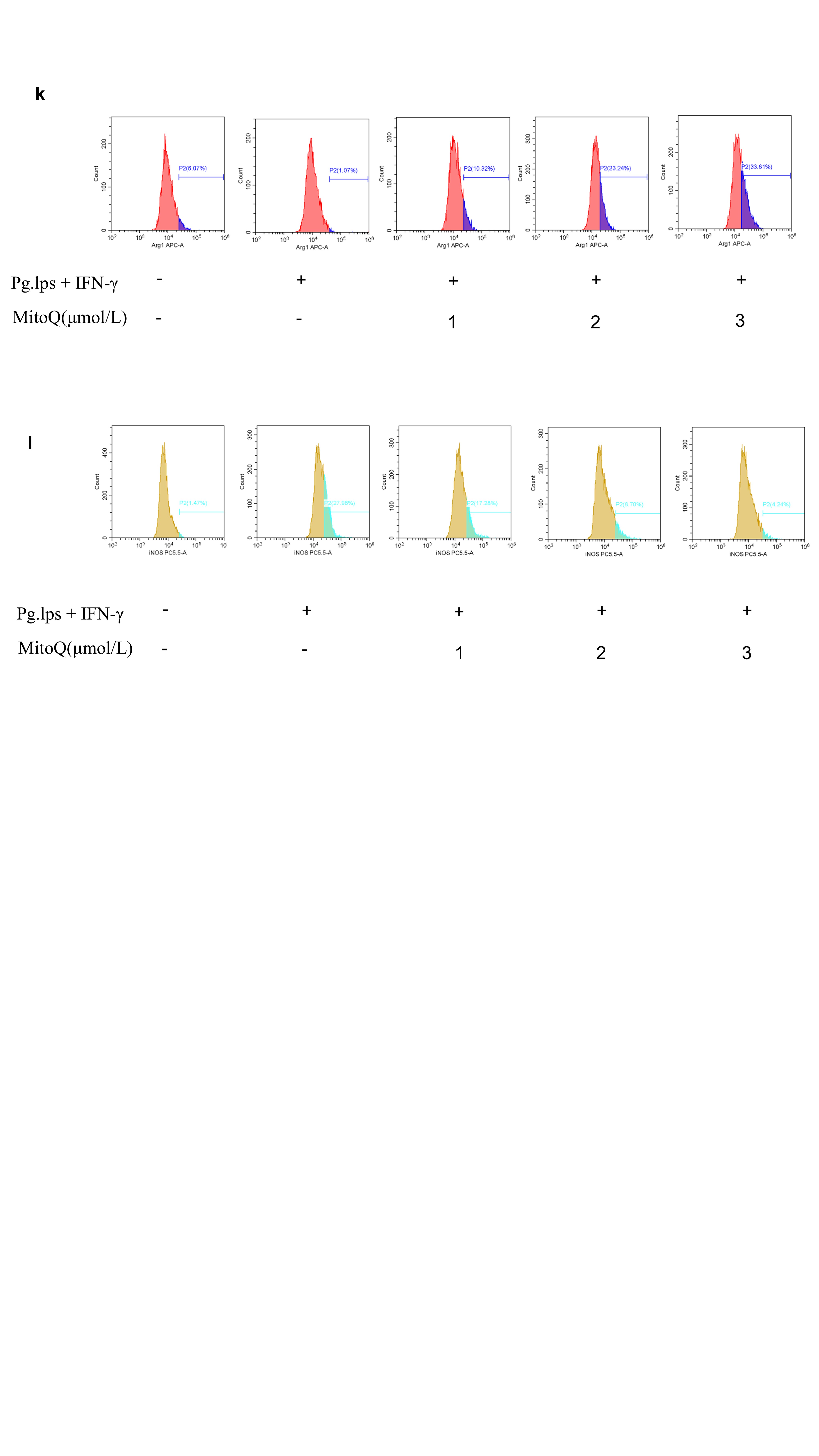


Figure S5. **a** Representative images of TMRM staining (scale bar = 100 um). **b** Semi-quantitative analysis of immunofluorescence for TMRM staining. **c** Representative images of Mitosox staining (scale bar = 100 um). **d** Semi-quantitative analysis of immunofluorescence for Mitosox staining. **e** The ATP content of RAW 264.7. **f** Representative images of DCFH-DA staining (scale bar = 100 um). **g** Semi-quantitative analysis of immunofluorescence for DCFH-DA staining. **h** The MDA content of RAW 264.7. **i** The SOD content of RAW 264.7. **j** The GSH content of RAW 264.7. **k** and **l** Flow cytometry analysis of the M1-related marker iNOS and the M2-related marker Arg-1. Data are shown as the mean± SEM and are representative of at least three independent experiments. *p < 0.05, **p < 0.01，***p < 0.001 and ****p < 0.0001 by T-test and one-way ANOVA followed by Tukey's post hoc test.


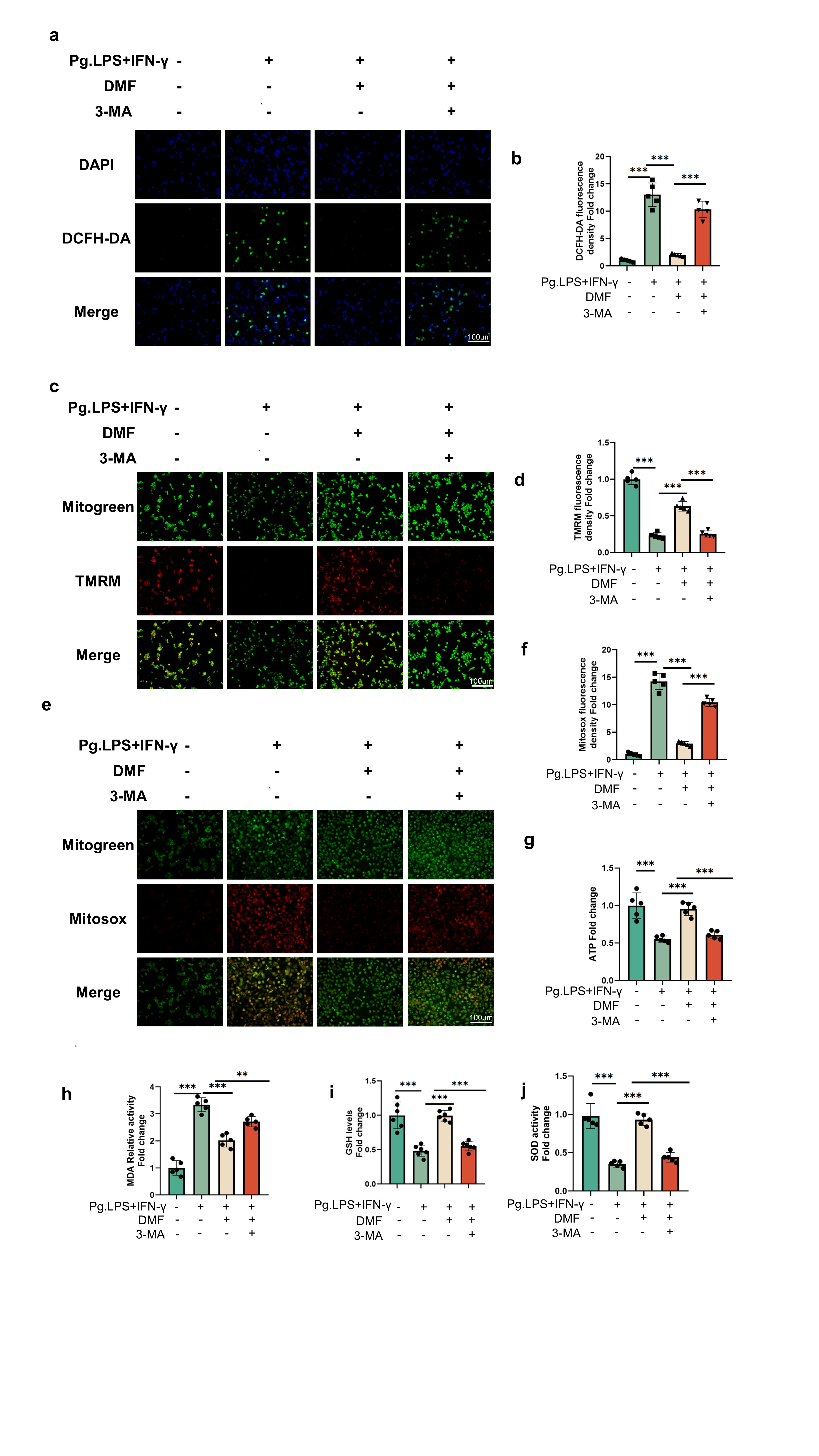


Figure S6. **a** Representative images of DCFH-DA staining (scale bar = 100 um). **b** Semi-quantitative analysis of immunofluorescence for DCFH-DA staining. **c** Representative images of tissue-resident memory staining (scale bar = 100 um). **d** Semi-quantitative analysis of immunofluorescence for TMRM staining. **e** Representative images of Mitosox staining (scale bar = 100 um). **f** Semi-quantitative analysis of immunofluorescence for Mitosox staining. **g** ATP content of RAW 264.7 cells. **h** Malondialdehyde content of RAW 264.7 cells. **i**Glutathione content of RAW 264.7 cells. **j** Superoxide dismutase content of RAW 264.7 cells. Data are shown as the mean±standard error of the mean and are representative of ≥3 independent experiments. *p < 0.05, **p < 0.01, and ***p < 0.001 using T-test and one-way analysis of variance followed by Tukey's post hoc test.


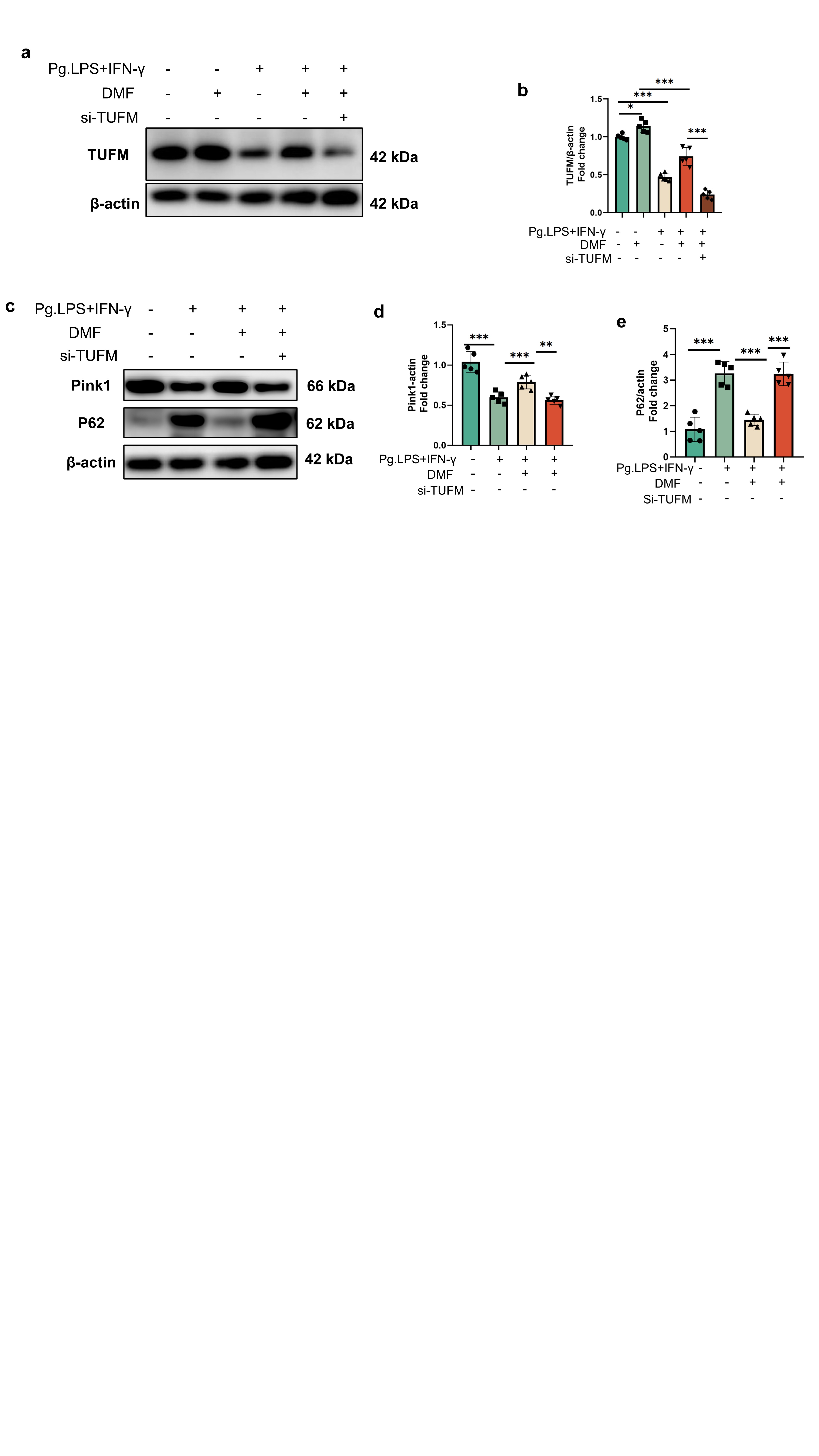


Figure S7. **a** Western blot band of TUFM expression in RAW 264.7 cells. **b** Level of iNOS and Argianse-1 relative to β-actin. **c** Western blot band of Pink1 and P62 expression in RAW 264.7 cells. **d**, **e** Level of Pink1 and P62 relative to β-actin. Data are presented as the mean±standard error of the mean and are representative of ≥3 independent experiments. *p < 0.05, **p < 0.01, and ***p < 0.001 using T-test and one-way analysis of variance followed by Tukey's post hoc test.


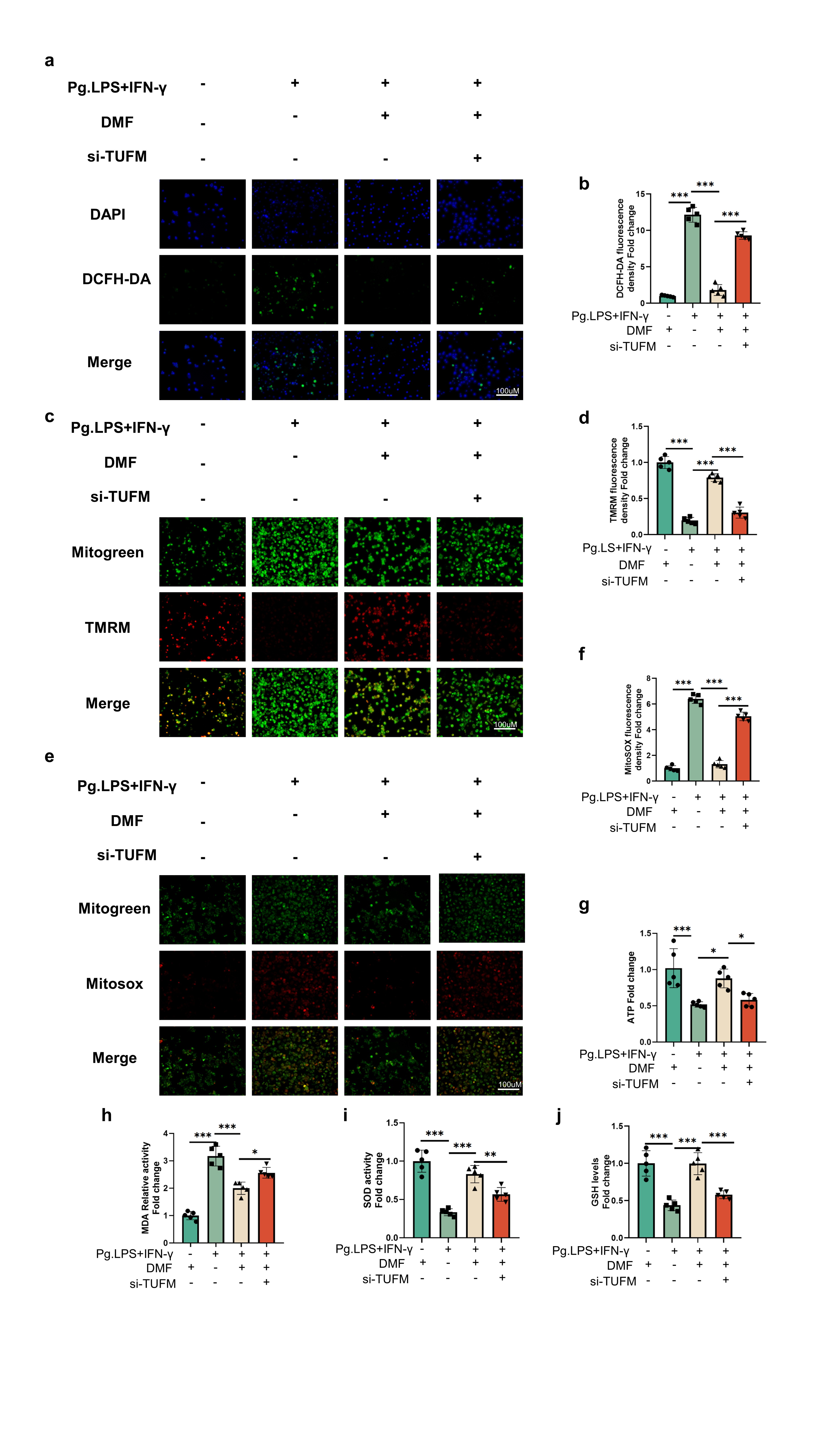


Figure S8. **a** Representative images of DCFH-DA staining (scale bar = 100 um). **b** Semi-quantitative analysis of immunofluorescence for DCFH-DA staining. **c** Representative images of tissue-resident memory staining (scale bar = 100 um). **d** Semi-quantitative analysis of immunofluorescence for TMRM staining. **e** Representative images of Mitosox staining (scale bar = 100 um). **f**Semi-quantitative analysis of immunofluorescence for Mitosox staining. **g** ATP content of RAW 264.7 cells. **h** Malondialdehyde content of RAW 264.7 cells. **i** Superoxide dismutase content of RAW 264.7 cells. **j** Glutathione content of RAW 264.7 cells. Data are presented as the mean± standard error of the mean and are representative of ≥3 independent experiments. *p < 0.05, **p < 0.01, and ***p < 0.001 using T-test and one-way analysis of variance followed by Tukey's post hoc test.


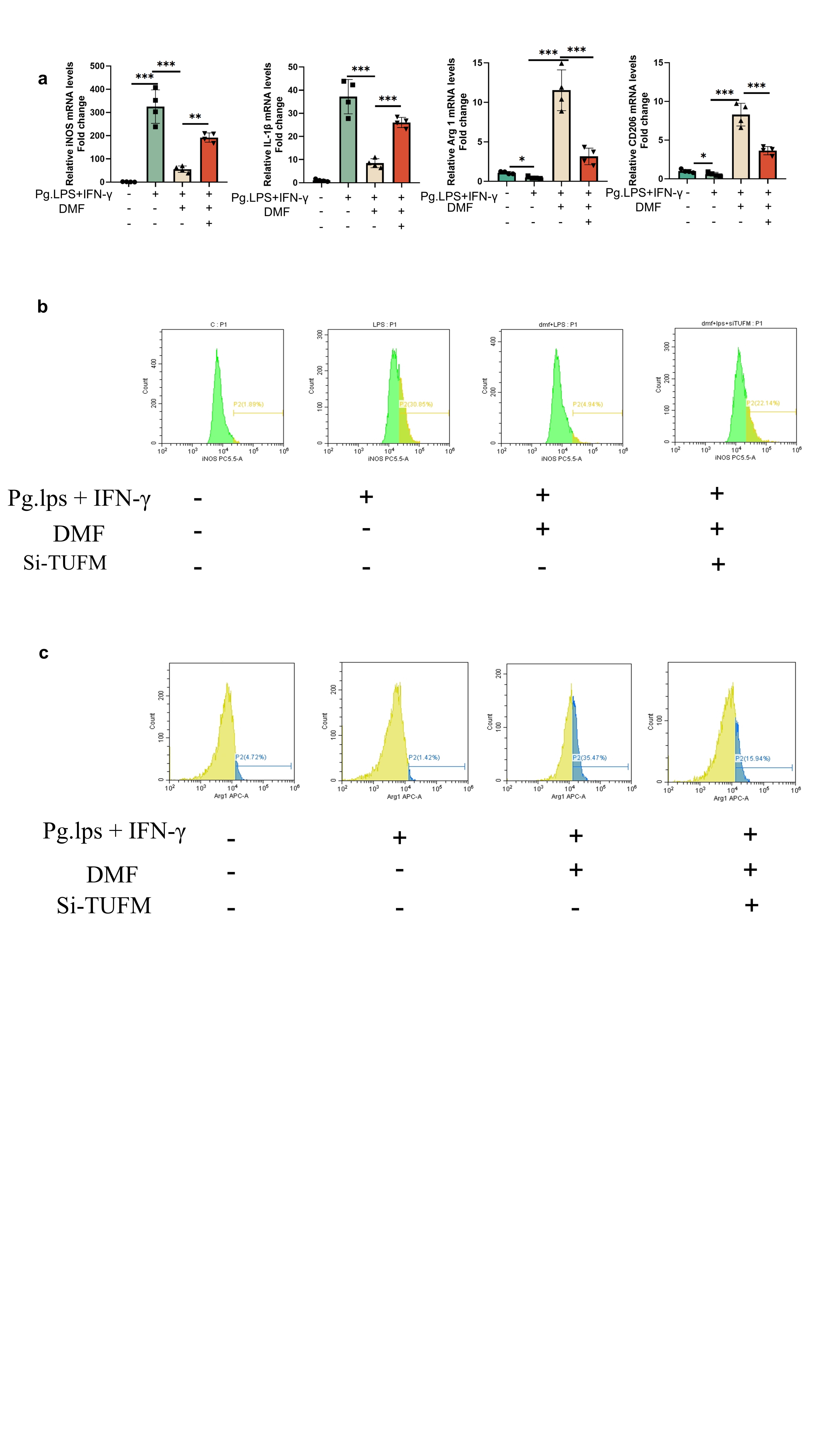


Figure S9. **a** Real-time polymerase chain reaction analysis of the gene expression of the M1-related iNOS and IL-1β and M2-related Arg1 and CD206. **b**, **c** Flow cytometry analysis of the M1-related marker iNOS and M2-related marker Arg-1. Data are presented as the mean±standard error of the mean and are representative of ≥3 independent experiments. *p < 0.05, **p < 0.01, and ***p < 0.001 using T-test and one-way analysis of variance followed by Tukey's post hoc test.

**Supplementary Table captions:**

Table S1: Primer sequences of mRNA

| Gene | Forward Primer (5'-3’) | Reverse Primer (5'-3’) |
| --- | --- | --- |
| IL-1β | TGAATTGGTCATAGCCCGCA | TCTCCTTCCTGTGCAAACTCT |
| iNOS | GTTCTCAGCCCAACAATACAA | GTGGACGGGTCGATGTCAC |
| CD206 | AGACGAAATCCCTGCTACTG | CACCCATTCGAAGGCATTC |
| Arg1 | GGAATCTGCATGGGCAACCTGTGT | CACCCATTCGAAGGCATTC |
| β-Actin | GCTGTCCCTGTATGCCTCTG | TTGATGTCACGCACGATTTCCC |
